# 821. Trends in the Use of Topical Corticosteroids for Granulation Tissue in Burn Wounds

**DOI:** 10.1093/jbcr/irag033.260

**Published:** 2026-04-06

**Authors:** Subhi Hakrush, Karen Shalata Hakrush, Ofir Ron, Noa Sabag, Dor Halpern, Inbal Alkalay, Gefen Bloom, Eliran Jacobi, Amir Abu Rabia, Basel Abu Ganem, Roy Grinberg, Rami Hanna, Eldad Silberstein, Jeremy Goverman, Yaron Shoham

**Affiliations:** Soroka University Medical Center, Be’er Sheva, HaDarom; Bnai Zion Medical Center, Be’er Sheva, HaDarom; Soroka University Medical Center, Be’er Sheva, HaDarom; Soroka University Medical Center, Be’er Sheva, HaDarom; Soroka University Medical Center, Be’er Sheva, HaDarom; Soroka University Medical Center, Be’er Sheva, HaDarom; Soroka University Medical Center, Be’er Sheva, HaDarom; Soroka University Medical Center, Be’er Sheva, HaDarom; Soroka University Medical Center, Be’er Sheva, HaDarom; Soroka University Medical Center, Be’er Sheva, HaDarom; Soroka University Medical Center, Be’er Sheva, HaDarom; Soroka University Medical Center, Nazareth, HaZafon; Soroka University Medical Center, Be’er Sheva, HaDarom; Massachusetts General Hospital, Brookline, MA; Soroka University Medical Center, Be’er Sheva, HaDarom

## Abstract

**Introduction:**

The presence of granulation tissue in burn wounds may delay healing and contribute to hypertrophic scarring and contractures. Common treatment methods include surgical excision, silver nitrate sticks, hypertonic saline, and laser ablation. Topical corticosteroids have been reported to suppress the inflammatory response underlying granulation tissue formation; however, evidence in burn wounds is limited and largely of low quality. In 2017, we surveyed burn care professionals at the 17th European Burns Association (EBA) Congress and found that topical steroid use for granulation tissue suppression was widespread despite the paucity of literature. To assess whether practice patterns and perceptions have evolved, we conducted a follow-up survey at the 21st EBA Congress in 2025.

**Methods:**

An anonymous questionnaire was distributed to attendees of the 21st EBA Congress (Berlin, 2025). Results were compared to responses from the original 2017 survey. Both surveys assessed respondent demographics, experience in burn care, annual patient volume, and perceptions of safety, efficacy, and complications associated with topical corticosteroid use in burns.

**Results:**

In 2017, 82 questionnaires were completed (74% physicians) with an average of 13.7 years of burn care experience and 300 patients treated annually; 88% practiced in Europe. Seventy-seven percent reported experience with topical steroids in burns, and all considered the treatment safe and effective. Most treated up to 50 burn patients with topical steroids annually, with only one (1.5%) reporting possible systemic side effects and 11% reporting rare infections (in <10% of patients they treated).

In 2025, 147 questionnaires were completed (76% physicians) with an average of 10.4 years of experience and 131 patients annually; 80% practiced in Europe. Sixty-seven percent reported experience with topical steroids in burns. Among these, almost all considered the treatment safe (98%) and effective (98%). Most treated up to 40 patients with topical steroids annually, while 19% treated >50 patients. Reports of systemic side effects (15%) and infections (20%) were more frequent than in 2017, though overall rates remained relatively low.

**Conclusions:**

The use of topical steroids for suppression of granulation tissue in burn care remains widespread in Europe. Compared with 2017, the proportion of professionals reporting experience slightly declined (77% to 67%), but confidence in safety and efficacy remains high. Reports of systemic side effects and infections were more common in 2025, underscoring the need for controlled clinical studies to further evaluate risks and benefits.

**Applicability of Research to Practice:**

Topical steroids continue to be perceived as a safe and effective adjunct for management of burn wound granulation tissue.

**Funding for the study:**

N/A.

